# An update on choroidal abnormalities and retinal microvascular changes in neurofibromatosis type 1

**DOI:** 10.1186/s13023-022-02369-8

**Published:** 2022-06-13

**Authors:** Fabiana Mallone, Luca Lucchino, Sandra Giustini, Alessandro Lambiase, Antonietta Moramarco

**Affiliations:** 1 Department of Sense Organs, Sapienza University of Rome, Policlinico Umberto I, Viale del Policlinico 155, 00161 Rome, Italy; 2 Department of Dermatology and Venereology, Sapienza University of Rome, Policlinico Umberto I, Rome, Italy

**Keywords:** Choroidal abnormalities (CAs), Neurofibromatosis type 1 (NF1), Diagnostic criteria, Hyperpigmented spots (HSs), Retinal microvascular abnormalities (RVAs)

## Abstract

Neurofibromatosis Type 1 (NF1) is a rare neurocutaneous disorder transmitted in an autosomal dominant fashion, mainly affecting the nervous system, the eye and skin. Ocular diagnostic hallmarks of NF1 include iris Lisch nodules, optic gliomas, orbital and eyelid neurofibromas, eyelid café-au-lait spots. In recent years, a new ocular sign represented by choroidal abnormalities (CAs) has been characterized in NF1. The CAs, identified with near-infrared reflectance, have been reported with a frequency of up to 100% in NF1, and have recently been added to the actual diagnostic criteria for NF1. The present Letter to the journal is intended to provide an update on features and clinical significance of CAs in NF1. Moreover, the relation with other ocular manifestations recently described in NF1 including hyperpigmented spots and retinal microvascular abnormalities is discussed.

## Dear Editor,

Neurofibromatosis type 1 (NF1), also termed von Recklinghausen disease, is a rare autosomal dominant multisystemic disorder, with complete penetrance and variable expressivity. It is caused by a mutation in the NF1 gene located on chromosome 17q11.2 that encodes for neurofibromin, a protein that controls cell growth and proliferation by regulating the proto-oncogene Ras; and it is 50% sporadic or inherited. The pathogenesis of NF1 involves neural crest-derived melanocytes, Schwann cells, prevertebral ganglion and sympathetic neurons. The disease can affect nearly all organ systems in the body. NF1 main characteristics are the appearance of various cutaneous, ocular and neurological manifestations, and an increased susceptibility to develop multiple benign and malignant tumors. The disease can also present as mosaicism, also called segmental NF1. In these cases, NF1 preserves the usual characteristics but is localized only in a body segment.

The eye and adnexa are frequently involved in NF1; some ocular manifestations including iris Lisch nodules, optic gliomas, orbital and eyelid neurofibromas, eyelid café-au-lait spots, are diagnostic hallmarks in NF1.

**Fig. 1:**
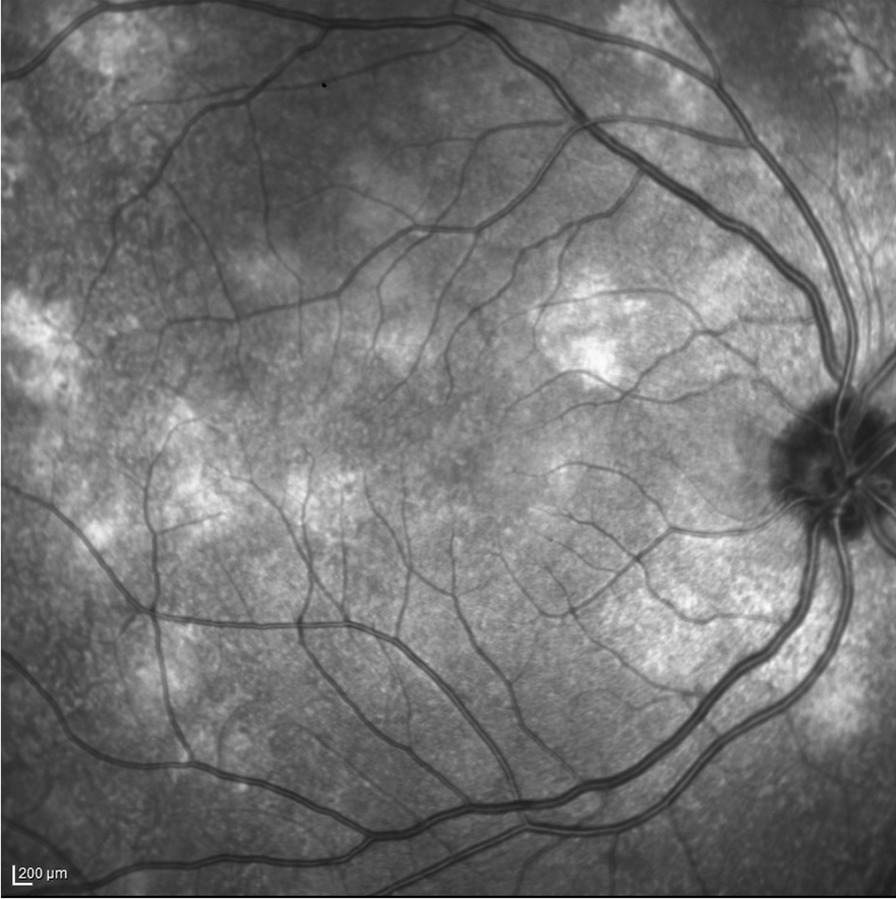
NIR-OCT image showing hyperreflective, patchy CAs in a NF1 patient

**Fig. 2:**
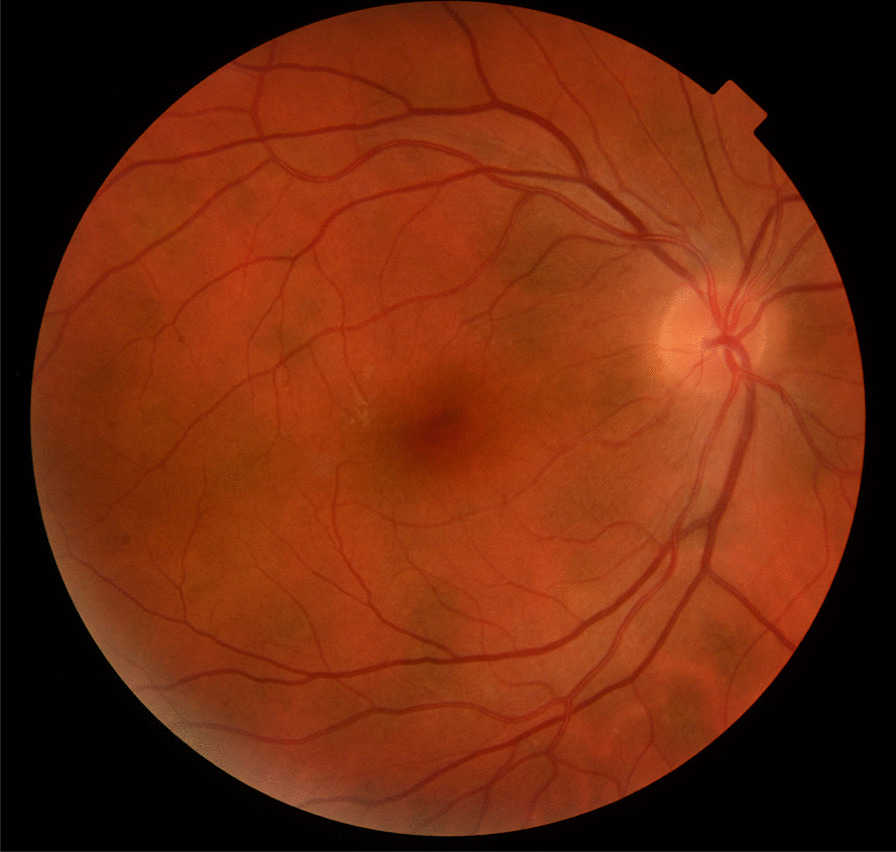
Color fundus photography showing rounded, HSs with blurred margins in NF1

**Fig. 3:**
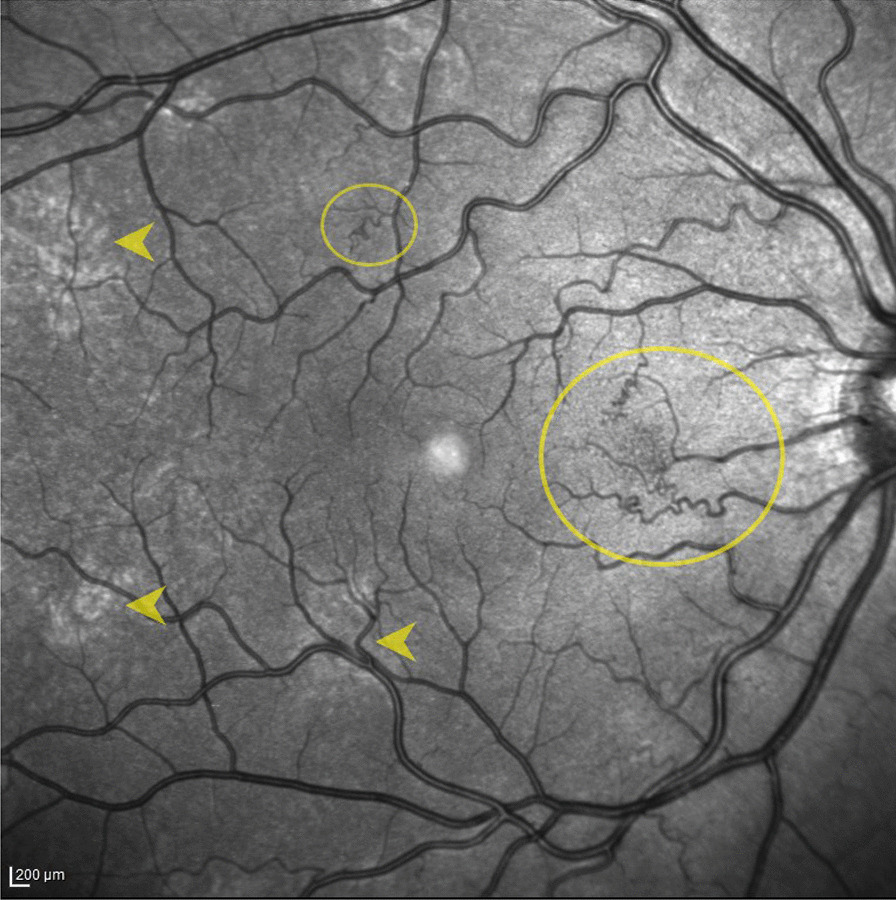
NIR-OCT representative image of well-defined, small, tortuous RVAs arising from small tributaries of the retinal veins within the main vascular arcades in NF1. Circles outline the RVAs while arrows indicate the CAs

In the last years, new manifestations including choroidal abnormalities (CAs), hyperpigmented spots (HSs) and retinal vascular abnormalities (RVAs), have been described in the ocular system in NF1, due to recent progress in multimodal imaging in ophthalmology (Figs. 1, 2, 3) [1–7].

Following a revision by an international consensus of experts, CAs have recently been added to the actual diagnostic criteria for NF1 based on their high specificity and sensitivity [8]. Furthermore, the presence of CAs allows to differentiate NF1 from Legius syndrome, which shows phenotypic overlap to NF1 [8, 9].

In the revised diagnostic criteria for NF1, CAs are not included as a separate criterion, but rather introduced as an alternative to the presence of iris Lisch nodules since isolated ophthalmologic findings, even if bilateral, are likely to reflect mosaic NF1 rather than constitutional NF1 [8]. Specifically, the patient is assigned 1 diagnostic criteria point in the presence of both CAs and iris Lisch nodules but he is still assigned 1 diagnostic criteria point also in the presence of either CAs or iris Lisch nodules. This is to avoid that, in the presence of both CAs and iris Lisch nodules, the patient is assigned 2 diagnostic criteria points and a diagnosis of NF1 is made, when there exists a possibility for it to be segmental NF1 [8]. 

CAs are known as ovoid bodies consisting of proliferating Schwann cells, neural crest-derived melanocytes and ganglion cells around axons arranged in lamellar patterns [10, 11]. Based on these features, CAs were attributed to hamartomatous lesions similar to the iris Lisch nodules, both showing the same embryological origin from the neural crest [12].

In previous studies, CAs were reported having higher prevalence (64–98%) in NF1 compared to iris Lisch nodules (41–86%), the latter considered to date the most frequent diagnostic ocular sign in NF1 [2, 3, 13–16]. Notably, the frequency of manifestation of approximately 100% of CAs is similar or second only to café-au-lait spots (98%), the most frequent diagnostic criterion in NF1 [2, 13, 14, 17]. In addition, CAs showed earlier age of presentation in NF1 compared to iris Lisch nodules with reported percentages of 64–95% and 41–52%, respectively, in the pediatric population [2, 13–16]. Furthermore, higher percentages (14–37%) of patients with NF1 were reported to have CAs but no iris Lisch nodules, while only a few patients (2.5–16%) had iris Lisch nodules in the absence of CAs [2, 13, 14, 16]. 

These observations may suggest that CAs are of higher diagnostic importance than iris Lisch nodules and support the inclusion of both CAs and iris Lisch nodules as relevant signs in the revised diagnostic criteria for NF1.

In the CAs, the proliferation of choroidal cell types causes a patchy choroidal thickening, resulting in a strong absorption and a subsequent backscattering of near-infrared light through the high content of melanin.

Recently, the spectral domain optical coherence tomography (SD-OCT) in near-infrared reflectance (NIR) modality, a non-invasive tool, has enabled superior visibility of CAs. Specifically, CAs appear as bright, patchy nodules on SD-OCT in NIR mode.

CAs are fully asymptomatic and undetectable with conventional ophthalmoscopic examination or by means of autofluorescence and fluorescein angiography. Indocyanine green angiography, at a wavelength similar to that of NIR, proved to be helpful in recording hypofluorescent patches corresponding to CAs in NF1, but it is an invasive diagnostic tool.

The hyperreflectivity of the CAs on NIR-OCT is attributable to a hyperactivity of the melanosomes and/or to an increase in the number of melanocytes in the choroid [18].

In agreement, CAs were reported to have predominant distribution within the main vascular arcades, based on greater thickness of the choroid and higher proportion of melanocytes in this area [19].

More recently, optical coherence tomography angiography (OCTA) studies demonstrated hyper-flow areas of deep choroid corresponding to the bright patches of CAs on NIR imaging [20].

Recent findings from our group reported that CAs may have different level of extension in the deeper choroid and different degree of pigmentation, reaching the level of visibility at fundus examination only in a minority of cases [3]. Specifically, as assessed through different wavelengths on ultra-wide field (UWF) scanning laser ophthalmoscopy and SD-OCT imaging, the most pigmented and inward extended CAs were visible as HSs at fundus examination, representing a new finding in NF1 [3].

This explains the different frequency of presentation of CAs and HSs, showing percentages of nearly 97% versus 24%, respectively, from our previous studies [3]. HSs appear as rounded, hyperpigmented areas with blurred margins at color fundus photography, with predominant distribution to the posterior pole similar to CAs location [3].

CAs are widely regarded as purely morphological alterations, which do not cause sensory abnormalities [2, 21]. However, their effect on surrounding retino-choroidal vasculature is yet to be established.

Interestingly, our group reported the association between HSs and NF1-related RVAs [3]. Also, previous studies investigated the relationship between CAs and overlying retinal microvascular changes in patients with NF1 [22].

RVAs, well-defined, small, tortuous retinal vessels arising from small tributaries of the retinal veins, constitute an ocular feature of recent observation in NF1 [1, 23]. Three different vascular patterns, ranging from a simple involvement to more complex manifestations, are known in literature: simple vascular tortuosity, corkscrew retinal vessels’ configuration and finally the moyamoya-like appearance [1].

In all cases, abnormal vessels occur in the superficial vascular plexus (SCP) from OCTA studies, with associated crowded and congested capillary network of the deep vascular plexus in the majority of cases [23, 24]. Similar to CAs and HSs, RVAs are mainly reported at the posterior pole [1].

Recently, a topographical correspondence has been described between CAs and overlying corkscrew retinal vessels, with debate on different pathogenetic hypotheses [22, 25]. It was speculated that the development of RVAs could possibly be related to disease-related disorders of vasomotor nerve cells or secretion of angiogenic factors by the CAs in NF1 patients [22, 25]. Results from OCTA studies limited to case series, reported abnormal retinal vessels overlying CAs with low flow areas and reduced vessel density at the level of choriocapillaris in NF1 [20, 26].

In a case–control study, the group of Vagge et al. reported a significant increase in the vascular flow area of the SCP and associated reduced choroidal vascular flow area in patients with NF1. These findings were attributed to a pathological redistribution of the vascular flow caused by the presence of CAs [27].

The results from these studies support the hypothesis that CAs compression and related blood flow redistribution could possibly have a role in the development of RVAs, however, this needs to be clarified in further work.

In conclusion, this Letter to the journal aims to provide insights on the features and clinical significance of CAs, a new diagnostic criterion for NF1, and emphasizes the importance for physicians of obtaining SD-OCT NIR imaging when evaluating patients with suspected NF1 in the clinic. Moreover, the strict relation between CAs and other ocular manifestations recently described in NF1 including HSs and RVAs was discussed. These new findings highlight the relevance of ocular involvement in NF1 disease, and this is expected to encourage further efforts in this field of clinical research.

## Data Availability

The data used to support the findings of this study are available from the corresponding author upon request.
